# Design and Implementation of the Hepatorenal Fibrocystic Disease Core Center Clinical Database: A Centralized Resource for Characterizing Autosomal Recessive Polycystic Kidney Disease and Other Hepatorenal Fibrocystic Diseases

**DOI:** 10.3389/fped.2017.00080

**Published:** 2017-04-20

**Authors:** Bakri Alzarka, Hiroki Morizono, John W. Bollman, Dongkyu Kim, Lisa M. Guay-Woodford

**Affiliations:** ^1^Division of Nephrology, Children’s National Health System, Washington, DC, USA; ^2^Center for Genetic Medicine, Children’s National Health System, Washington, DC, USA; ^3^Center for Translational Science, Children’s National Health System, Washington, DC, USA

**Keywords:** autosomal recessive polycystic kidney disease, congenital hepatic fibrosis, hepatorenal fibrocystic disease, ciliopathies, Cerner Health Facts^®^, REDCap, i2b2

## Abstract

Autosomal recessive polycystic kidney disease (ARPKD) and other hepatorenal fibrocystic diseases (HRFD) are relatively rare recessive disorders that constitute an important set of childhood nephropathies. Little is known about fundamental pathogenesis, and advances toward clinical trials will require well-characterized patient cohorts and the development of predictive and prognostic biomarkers. Such studies in rare diseases require greater collaboration than the efforts in common diseases where large patient repositories can be built at a single site. For the HRFD, clinical and translational research studies would be well served by centralized case accrual that coordinates collection of clinical data, biospecimens (DNA and tissues), and genetic information. As a part of the NIH-funded Hepatorenal Fibrocystic Disease Core Center, we have established a web-accessible portal to enroll patients with ARPKD and other HRFD and compile baseline and longitudinal clinical information in a REDCap-based clinical database. This central database is structured to collect clinical data from patients throughout the Americas (North, Central, and South). By using informatic analyses, we have defined the first data-driven estimates of ARPKD-related neonatal mortality, as well as the incidence and prevalence of this disease. These data indicate that while ARPKD is a rare disorder, there are hundreds of patients potentially available for deep clinical phenotyping in the United States alone. The centralization and sharing of clinical information and biomaterials from ARPKD and other HRFD patients hold the potential to accelerate progress in understanding disease pathways. Once the database is mature, the well-characterized patient cohorts will provide an important resource for developing clinical trials to evaluate new targeted therapeutic interventions in this spectrum of disorders.

## Introduction

Multiple single gene disorders that are characterized by renal cystic disease and extrarenal manifestations involve proteins that are critical in the structure/function of the primary apical cilium (1). As a result, this broad class of disorders is increasingly referred to as the “ciliopathies” (2). A subset of the ciliopathies is specifically characterized by fibrocystic disease of the kidney and dysgenesis of the portobiliary tract (congenital hepatic fibrosis and/or Caroli disease), prompting a refined description of these disorders as hepatorenal fibrocystic diseases (3, 4). Autosomal recessive polycystic kidney disease (ARPKD) is considered to be the flagship disorder in this new phenotypic subclassification (5, 6), which also includes rarer recessive disorders such as nephronophthisis (MIM 256100), Joubert syndrome (MIM 213300), Bardet–Biedl syndrome (MIM 209900), Meckel–Gruber syndrome (MIM 249000), and oro-facial-digital syndrome I (MIM 311200). The latter disorders have variable degrees of renal cystic disease and can be associated with congenital hepatic fibrosis and/or Caroli disease.

This family of hepatorenal fibrocystic diseases (HRFD) constitutes an important set of childhood nephropathies due to the associated high disease burden and early mortality. For example, in ARPKD, affected children typically present *in utero* with enlarged, echogenic kidneys, as well as oligohydramnios secondary to poor urine output. Significant perinatal mortality results from severe pulmonary hypoplasia and secondary respiratory insufficiency. Among survivors, the clinical phenotype can be quite variable with systemic hypertension, renal insufficiency, and portal hypertension due to portal tract fibrosis (7). The rarity of ARPKD and other disorders in the HRFD spectrum diseases presents great challenges for researchers trying to characterize and understand them sufficiently to develop strategies for targeted intervention (8).

Despite the identification of *PKHD1* and other genes involved in the HRFD disorders, pathogenic mechanisms remain poorly understood. Factors that modulate disease expression are not well defined, and there remains wide-spread pessimism about prognosis for many of these patients. To optimize clinical decision-making and develop more effective targeted therapies, the natural history of ARPKD and other HRFD needs to be better characterized; the impact of recent advances in neonatal care and pediatric renal replacement therapy on patient survival must be systematically evaluated; and predictive/prognostic markers must be developed and validated to inform the design of new clinical trials.

Research in such rare diseases requires greater collaboration than the efforts in common diseases where patient resources are routinely available and large repositories can be built locally. For the HRFD, experimental studies would be well served by coordinated case accrual with integration of clinical data, biospecimens (DNA and tissues), and genetic information from individual patients through a unique identifier. The centralization and sharing of clinical and genetic information, as well as biomaterials, would provide a critical resource for accelerating research progress.

Our group has previously described the ARPKD clinical experience in North America (9). We developed and analyzed a longitudinal clinical database of ARPKD patients who had been recruited from pediatric nephrology centers in the United States and Canada. However, the study relied on individual centers to obtain institutional review board (IRB) approval for site participation, as well as to consent local patients, extract data from medical records, and submit data entry forms. These requirements presented an often insurmountable burden to the contributing physicians. To overcome these challenges and still maintain the electronic medical record as a validated data source, a different and more time efficient approach was needed.

Therefore, with funding from the National Institutes of Health, we established the Hepatorenal Fibrocystic Disease Core Center (HRFDCC),^1^ an interdisciplinary research center with a particular focus on ARPKD, but also including other single gene disorders that comprise the HRFD spectrum. Here, we describe the design of the HRFDCC Clinical Database, a centralized platform that allows patients/families parents to enroll their children through an online portal, thus streamlining patient recruitment and obviating the need for contributing clinicians to obtain local IRB approval. The HRFDCC Clinical Database is designed to capture the clinical variability within and among this set of disorders and ultimately to serve as a resource of well-phenotyped patients for future interventional studies.

## Feasibility Analyses

As a group, the HRFD are relatively rare. The estimated incidence of each disorder is largely derived from limited clinical and genetic studies. There are no prevalence estimates available in the literature. To ascertain the size of the potential cohort available for recruitment to the HRFDCC Clinical Database, we performed a systematic review using the national Cerner Health Facts^®^ (HF) Database, which captures and stores deidentified, longitudinal electronic health record (EHR) patient data from more than 500 facilities in the United States and then aggregates and organizes it into consumable data sets. For these analyses, we focused on estimating the US-based incidence and prevalence of ARPKD, the most common of the HRFD, using data for the 5 years (2010–2014) before the ICD-10 coding system was implemented (October 2015).

In brief, we developed an Oracle-based local instance of the HF Database and ascertained the number of unique patients (age 0–29 years) with the ARPKD ICD-9 diagnosis code (753.14) between January 2010 and December 2014. We determined the annual number of unique ARPKD newborns (<1 month of age) and the annual number of all newborns (<1 month of age) in the HF Database. We also determined the annual number of US births from the National Vital Statistics Report.^2^ On the basis of HF data set, we calculate a 79% survivor rate among ARPKD neonates. As shown in Table 1, our analyses indicate that the annualized incidence of ARPKD is 1:26,485 live births, from which we estimate that there are ~120 new ARPKD neonatal survivors per year in the United States. This calculated incidence corresponds to the reported incidence of 1:20,000 live births derived from previous genetic studies (10). We used a similar strategy to calculate the annualized prevalence of ARPKD (Table 2). On the basis of these analyses, we propose a conservative estimate of approximately 1,500 ARPKD patients (age 0–29 years) living in the United States.

**Table 1:** **Estimating the incidence of ARPKD using Cerner Health Facts^®^ Data**.

	HF ARPKD patients (<1 month)	HF (<1 month)	ARPKD incidence in HF^a^	NVS	HF/NVS (%)	ARPKD annual new patients^b^	Adjusted ARPKD annual new patients^c^
2010	9	279,902	1:31,100	3,999,386	7.00	129	102
2011	13	322,416	1:24,801	3,953,590	8.16	159	126
2012	9	297,885	1:33,098	3,952,841	7.54	119	94
2013	11	295,432	1:26,857	3,932,181	7.51	146	115
2014	15	313,988	1:20,933	3,988,076	7.87	191	151
AVG	11	301,925	1:26,485	3,965,215	7.61	150	118

*^a^HF ARPKD < 1 month/HF < 1 month*.

*^b^HF ARPKD < 1 month/HF/NVS*.

*^c^Based on 79% ARPKD neonatal survivor rate in HF data set*.

*ARPKD, autosomal recessive polycystic kidney disease; HF, Health Facts^®^ Dataset (using discharge data); NVS, National Vital Statistics data*.

**Table 2:** **Estimating the prevalence of ARPKD using Cerner Health Facts^®^ Data**.

	HF ARPKD patients (0–29 years)	HF (0–29 years)	US population (0–29 years)^a^	HF/US population (0–29 years) (%)	ARPKD patients (0–29 years)^b^	Annualized prevalence (per 100,000)
2010	55	6,228,323	126,025,383	4.94	1,113	0.88
2011	78	7,140,758	126,252,347	5.66	1,379	1.09
2012	89	6,858,426	126,452,548	5.42	1,641	1.30
2013	76	6,996,558	127,084,385	5.51	1,380	1.09
2014	112	7,727,920	127,326,557	6.07	1,845	1.45
AVG	82	6,990,397	126,628,244	5.52	1,485	1.17

*^a^From NVS data set*.

*^b^Corrected for US population*.

*ARPKD, autosomal recessive polycystic kidney disease; HF, Health Facts^®^ Dataset (using discharge data); NVS, National Vital Statistics data*.

We believe that our analyses provide reasonable estimates of incidence, annual neonatal mortality rate, and the prevalence for ARPKD. Moreover, these data indicate that for this rare disease, there are hundreds of patients available for deep clinical phenotyping in the United States alone.

## Database Development

### Inclusion Criteria

The HRFDCC Clinical Database focuses on patients aged 0–35 years who have renal imaging consistent with ARPKD or another HRFD (9) and one or more of the following findings.

- Mutations detected in the *PKHD1* gene or other HRFD genes;
- Clinical/imaging evidence of biliary fibrosis and/or portal hypertension;
- Hepatic histopathology consistent with ductal plate malformation;
- Family history consistent with autosomal recessive inheritance.

Of note, the classic renal sonographic findings in ARPKD involve enlarged, diffusely echogenic kidneys with poor corticomedullary differentiation. With the development of high-resolution ultrasound, the sonographic pattern of ARPKD, particularly in older infants and children, can include scattered medullary and cortical macrocysts (due to dilated collecting ducts becoming enclosed cysts that enlarge) that are superimposed on the background of diffuse hyperechogenicity (11).

Participants are excluded if they have autosomal dominant polycystic kidney disease (ADPKD) or renal dysplasia, with or without urinary tract malformations.

### Data Collection Instruments

Electronic case report forms (CRFs) were developed to collect initial patient data and follow-up information at yearly intervals. These CRFs have built-in edit checks tagged to each data field to minimize the risk of missing or duplicated data. The Initial Visit CRF records demographic characteristics (e.g., gender and race), date of birth, date of diagnosis, specific clinical diagnosis, last contact, and death (if applicable). Additional information includes family history and parental consanguinity. Perinatal data are also collected, e.g., whether the diagnosis was made prenatally, and if so, the diagnostic modality used, e.g., sonography or genetic analysis.

The Follow-up Visit CRF records interval changes and the date of last contact. When applicable, the date and cause of death are specified. The follow-up data entries are planned to occur annually.

Each CRF (initial and annual follow-up) includes the following.

- Patient data: age, body measurements;
- Pulmonary status: ventilation, supplemental oxygen;
- Patient laboratory data;
- Renal data: clinical issues (e.g., hypertension, urinary tract infection), radiological findings, biopsy results;
- Renal replacement therapy: dates (initiation of dialysis or transplantation), dialysis modality, donor source (living-related donor versus cadaveric), and the status of the graft at last contact;
- Hepatic data: symptoms, radiological findings, biopsy results. If appropriate, the date of liver transplantation and donor source;
- Medication data: start and end date, dose;
- Other therapeutic interventions: supplemental feeding (e.g., nasogastric or gastric tubes), endoscopy, surgical procedures (e.g., portal vein shunting or splenectomy);
- Related data: whether genetic testing has been performed; availability of biosamples.

### Database Design

We leveraged the previously reported North American ARPKD Database to establish the HRFDCC Clinical Database as a secure, HIPAA-compliant, web-based portal for clinical data entry.^3^ The database is built on the REDCap platform, a widely used, secure web application for building and managing online databases^4^ (12). REDCap has built-in tools that can validate data, check data consistency across variables, and perform “intelligent” form processing by altering form navigation based on prior responses. The application permits easy generation of data reports, graphical representations, and descriptive statistics by allowing designated users to export data to Excel, SAS, Stata, R, or SPSS for analysis. Custom reports can also be generated in which the report is filtered to specific fields, records, or events using an extensive array of tools that allow the user to retrieve the precise data set of interest. Additional fields can be added to the custom reports and filtered by adding filter logics. The reports created can be modified or deleted at any time.

The HRFDCC Clinical Database implements an IRB-approved algorithm for clinical data entry that obviates the need for the contributing physician to obtain local IRB approval. In brief, the patient/family contacts the website, indicates interest in participating in the research study, and downloads the study informed consent and a Release of Medical Information Authorization form. The HRFDCC Clinical Database research nurse is automatically alerted by a system-generated e-mail, which provides the participant contact information. Prompted by this alert, the research nurse contacts the participant to review the informed consent and address questions about the study. The signed consent and the medical records can be returned to the study team by mail or FAX.

Upon receipt of the signed consent, a unique identifier is autogenerated for the participant. The HRFDCC Research Nurse Data extracts data from each corresponding medical record and enters these data into the password-restricted database using the participant’s unique identifier. The database is housed on a dedicated server at the Children’s National Health System and secured by communication channels that use https and Secure Socket Layer encryption. Therefore, the data in the servers are restricted to authorized personnel.

Built into the database design are the following features.

- Real-time mechanism for rules-based edit checking and data validation.
- Real-time alert mechanism to flag data of particular interest and notify appropriate individuals.
- Capability to generate summary reports about the cohort’s demographics and aggregate clinical and genetic data or other reports as requested.
- A search function, so that as the Database matures, researchers can mine the data using specific clinical and genetic queries.

The study has been approved by the CNHS IRB, and the consent is available in English, Spanish, Portuguese, and French, allowing recruitment of patients from North, Central, and South America.

### Informatics-Based Approach for Participant Recruitment

As a single-site demonstration project, we developed a computer algorithm to identify ARPKD patients who meet eligibility criteria for HRFDCC Clinical Database. The algorithm was built to run queries based upon the ARPKD ICD-9 code (753.14), using the i2b2 Clinical and Translational Research Informatics Data Warehouse, a resource developed by the Clinical and Translational Science Institute at Children’s National (CTSI-CN) as a repository of patient data from the Cerner EHR. The i2b2 query returned a cohort of 41 patients. With IRB approval, the medical record number was obtained for each patient. Manual EHR review revealed that 20 of 41 (49%) patients met the eligibility criteria for ARPKD. The remaining 21 (51%) patients had diagnoses ranging from ADPKD, renal dysplasia, multicystic dysplastic kidney, to mothers of affected fetuses.

## Current Status

### Cohort Characteristics

To date, 105 patients have met our inclusion criteria and been enrolled in the HRFDCC Clinical Database. Of these, 83 patients from across the United States have clinical, imaging, and/or histopathological data consistent with ARPKD. The mean age of the cohort is 12.0 years, with a mean age of 11.17 years in males and 12.52 years in females. Initial analyses summarized in Tables 3 and 4 demonstrate that (1) the gender distribution is relatively equivalent, consistent with an autosomal recessive disorder; (2) Caucasians represent the predominant ethnicity, as noted in previous reports (9, 13); (3) diagnosis was made prenatally in 41% of patients, primarily based on renal sonography; and (4) a family history was noted for 21.7% patients, primarily involving an affected sibling. In this cohort, 40% required mechanical ventilation at birth and 24% had chronic lung disease (as indicated by the need for supplemental oxygen). Hyponatremia was reported in 31.3%, primarily in the first few months of life. Hypertension was the most common comorbidity, reported in 72.3% of the cohort, with angiotensin-converting enzyme inhibitor agents being the most commonly used class of antihypertensive medications. As would be predicted in this older cohort, chronic kidney disease was common, with 57.8% having an estimated glomerular filtration rate (14) of less than 90 ml/min per 1.73 m^2^ and 22.9% requiring either dialysis or preemptive transplantation. Comorbidities related to periportal fibrosis included splenomegaly, varices with or without associated bleeding, and cholangitis. The frequency of these ARPKD-related morbidities largely reflect that in previous reports (9, 15, 16), indicating that even with the limited, family-directed patient enrollment in this database, the ARPKD cohort mirrors the phenotypic features described in previous cohorts.

**Table 3:** **Characteristics of the autosomal recessive polycystic kidney disease cohort in the Hepatorenal Fibrocystic Disease Core Center Clinical Database**.

Characteristics	Number (%)
**Gender**	
Male	48 (57.8)
Female	35 (42.2)
**Race**	
White	64 (77.1)
Black	7 (8.4)
Hispanic	8 (9.7)
Other	4 (4.8)
**Prenatal diagnosis**	
Yes	34 (41)
No	46 (55.4)
Unknown	3 (3.6)
**Status as of 12/01/2016**	
Alive	66 (79.5)
Dead	14 (16.9)
Unknown	3 (3.6)
**Family history of PKD**	
Yes^a^	18 (21.7)
No	58 (69.9)
Unknown	7 (8.4)

*^a^Affected sibling*.

**Table 4:** **Autosomal recessive polycystic kidney disease comorbidities and interventions**.

	Number (%)
**Neonatal ventilation**	
Yes	33 (40)
No	50 (60)
**Chronic lung disease**	
Yes	20 (24)
No	63 (67)
**Growth retardation**	
Yes	10 (12)
No	73 (88)
**Hyponatremia**	
Yes	26 (31.3)
No	57 (68.7)
**Hypertension**	
Yes	60 (72.3)
No	23 (27.7)
**eGFR (<90 ml/min per 1.73 m2)**	
Yes	48 (57.8)
No	35 (42.2)
**Dialysis**	
Yes	17 (20.5)
No	66 (79.5)
**Kidney transplant^a^**	
Yes	9 (10.8)
No	74 (89.0)
**Varices**	
Yes	10 (12)
No	73 (88)
**Variceal bleeding**	
Yes	6 (7.2)
No	77 (92.8)
**Cholangitis**	
Yes	6 (7.2)
No	77 (92.8)
**Splenomegaly**	
Yes	16 (19.3)
No	67 (80.7)
**Liver transplant**	
Yes	1 (1.2)
No	82 (98.8)

*eGFR, estimated glomerular filtration rate*.

*^a^Kidney transplant: of the nine patients, seven were previously on dialysis and are counted in the dialysis cohort and two patients received a preemptive kidney transplant without prior dialysis*.

### Recruitment Limitations

Our experience to date indicates that many, but not all HRFD families, are interested in participating in clinical studies such as the HRFDCC Clinical Database. However, a significant subset is either not able, or interested in, completing our web-based enrollment protocol. Several factors appear to contribute, including (1) limited computer literacy, (2) language barriers, (3) delays in completing the informed consent process, (4) burden on the local physician—the initial database design required local physicians to complete the online data entry forms using the patient unique identifier, and (5) parental expectation that genetic analysis would be part of the protocol, with return of results to the family for clinical use. Our inability to provide free genetic testing has been a particularly significant limiting factor. This issue was further compounded by a concurrent NHGRI-sponsored study *Evaluation of Autosomal Recessive Polycystic Kidney Disease and Congenital Hepatic Fibrosis* that began enrolling in September 2003 and performed genetic testing as a part of the protocol.

We have taken a multipronged approach to address these issues, including (1) enhanced partnership with patient advocacy groups such as the Polycystic Kidney Disease Foundation to advertise the HRFDCC Clinical Database; (2) translation of the study protocol and informed consent into Spanish, French, and Portuguese; (3) enhanced central support to field family questions and assist with initial enrollment; and (4) centralized data entry using medical records obtained through a Release of Medical Information, thus obviating the burden on local physicians. In addition, we are working to improve the positive predictive value and the sensitivity of the computable algorithm for EHR-based patient discovery through a process of iterative i2b2 queries validated by manual record review. This approach has been used by others to rapidly and accurately identify a sickle-cell disease cohort within the EHR (17). We will then deploy this “computable phenotype algorithm” for use in centers with particular expertise in ARPKD to enhance the identification and recruitment of these patients to the HRFDCC Clinical Database.

A key issue that remains to be addressed is how best to incorporate genetic analysis into our protocol, both to improve enrollment and to allow analysis of potential genotype–phenotype correlations in this diverse cohort. Following the collaborative model developed between ARegPKD, the European ARPKD registry study (reviewed in this issue), and the German NEOCYST (Network for Early Onset Cystic Kidney Disease) Consortium, we are exploring mutually beneficial, cost-effective partnerships with the NIH-funded programs that are using next-generation sequencing technologies to accelerate the use of genetic analysis in clinical care (e.g., the Duke Task Force for Neonatal Genomics^5^).

In summary, we have established a REDCap-based clinical database to compile baseline and longitudinal clinical information on patients with ARPKD and other HRFD. Our informatic analyses indicate that while ARPKD is a rare disorder, there are hundreds of patients potentially available for deep clinical phenotyping in the United States alone. Once the database is mature, well-characterized HRFD cohorts will provide an important resource for designing clinical trials to evaluate new, targeted therapeutic interventions in this spectrum of disorders.

## Ethics Statement

The study has been approved by the CNHS Institutional Review Board, and the consent is available in English, Spanish, Portuguese, and French, allowing recruitment of patients from North, Central, and South America.

## Author Contributions

BA interpreted the data/drafted and revised the article. DK, HM, and JB developed and performed the informatics analyses. LG-W conceptualized the article, provided guidance with the content of the article, and critically reviewed and revised the article. All authors approved the final article as submitted.

## Conflict of Interest Statement

The authors declare that the research was conducted in the absence of any commercial or financial relationships that could be construed as a potential conflict of interest.

## Footnotes

^1^www.uab.edu/medicine/hrfdcc.

^2^http://www.cdc.gov/nchs/products/nvsr.htm.

^3^http://www.arpkdstudies.uab.edu/.

^4^http://www.project-redcap.org.

^5^https://www.dukegenes.org.
